# Disentangling the impacts of outcome valence and outcome frequency on the post-error slowing

**DOI:** 10.1038/srep08708

**Published:** 2015-03-03

**Authors:** Lijun Wang, Dandan Tang, Yuanfang Zhao, Glenn Hitchman, Shanshan Wu, Jinfeng Tan, Antao Chen

**Affiliations:** 1 Key Laboratory of Cognition and Personality of Ministry of Education, Faculty of Psychology, Southwest University, Chongqing 400715, China

## Abstract

Post-error slowing (PES) reflects efficient outcome monitoring, manifested as slower reaction time after errors. Cognitive control account assumes that PES depends on error information, whereas orienting account posits that it depends on error frequency. This raises the question how the outcome valence and outcome frequency separably influence the generation of PES. To address this issue, we varied the probability of observation errors (50/50 and 20/80, correct/error) the “partner” committed by employing an observation-execution task and investigated the corresponding behavioral and neural effects. On each trial, participants first viewed the outcome of a flanker-run that was supposedly performed by a ‘partner’, and then performed a flanker-run themselves afterwards. We observed PES in the two error rate conditions. However, electroencephalographic data suggested error-related potentials (oERN and oPe) and rhythmic oscillation associated with attentional process (alpha band) were respectively sensitive to outcome valence and outcome frequency. Importantly, oERN amplitude was positively correlated with PES. Taken together, these findings support the assumption of the cognitive control account, suggesting that outcome valence and outcome frequency are both involved in PES. Moreover, the generation of PES is indexed by oERN, whereas the modulation of PES size could be reflected on the alpha band.

Response monitoring is one crucial aspect in performance control. After detecting an error, the response monitoring system will initiate remedial actions to avoid future aversive outcomes. Post-error slowing effect (PES) is an example of such a remedial action, manifested as reaction time slowing following errors^1^. Although many studies have focused on the underlying neural mechanisms of PES, no agreed conclusions regarding the exact neural system mediating this effect has been reached^2^,^3^,^4^,^5^,^6^,^7^.

The cognitive control account assumes that the slowing is specific to errors. Error signals initiate cognitive control mechanisms to improve subsequent performance by activating the anterior cingulate cortex (ACC)^2^,^3^, in which, error signals are associated with a subsequent intensification of top-down control. Thus, participants will take a more conservative response strategy (slower and more accurate) after error commission to rectify aversive outcome. A challenge to this account is recently put forth by Notebaert and his colleagues^5^. They suggest that the slowing depends on the event frequency. Infrequent outcomes draw away the attentional resources, and then participants need to take more time to reorient the subsequent trials. Thus, PES is just a special case, even when the correct responses are infrequent events, post-correct slowing will be observed^5^,^8^.

Aforementioned controversies invite a systematic investigation how the outcome valence and outcome frequency impact on PES. In the present study, to achieve predetermined outcome frequency, error rates have to be effectively manipulated. Since errors usually occur at a low probability in the daily life, even in the laboratory environment, a number of studies have provided converging evidence that monitoring one's own and other's performance shares a common neural circuit^9^,^10^,^11^,^12^,^13^. This neural process can be interpreted by the simulation theory, which claims that other's internal states can be gained by internally simulating the processes that occur in another person's brain^14^,^15^,^16^. Moreover, several studies have affirmed that behavioral adjustments that are triggered by other-generated errors and by one's own errors are the same, indicating a slowing effect occurs following observation errors^16^,^17^. More importantly, error rates can be intentionally manipulated by employing the observation errors, the problem that the laboratory tasks are often too simple to produce enough errors is therefore resolved without increasing the task difficulty. Thus, an observation-execution Flanker task (Fig. 1) was employed in the present study. During each trial, participants first viewed the outcome of a flanker-run that was supposedly performed by a ‘partner’ in another room, and then performed a flanker-run themselves afterwards. In the Flanker task, participants were required to respond to the central target stimuli while ignoring the flanking stimuli that might suggest the same response with the target (congruent trials) or an opposite response to the target (incongruent trials)^18^,^19^.

In the last decades, studies employing electroencephalographic (EEG) techniques usually take the time- and phase-locked event-related potential (ERP) approach to investigate the neural correlates of error processing^20^,^21^. ERP approaches average epochs of EEG data associated with events of interest (target stimuli or response outcomes) to reveal the neural activities elicited by the events^22^,^23^. Therefore, the information that is contained in noise unrelated to the events will be largely cancelled out. However, alternative time- but not phase-locked time-frequency analysis extracts the power of multiple frequency bands in the ongoing EEG as a function of time^22^,^24^. This method can help to fully utilize the EEG data, including the phase-inconsistent part of the EEG that is invisible in the ERP. Therefore, to elucidate the specific contributions of outcome valence and outcome frequency in the generation of PES, we investigated the neural dynamics of outcome valence and outcome frequency using EEG, including the ERP and time-frequency approaches.

Previous studies identified two typical ERP components by averaging a set of epochs time-locked to the error trials: error-related negativity (ERN)^21^,^25^ and error positivity (Pe)^26^,^27^. ERN is a negative-going wave appearing about 50–100 ms after errors over frontocentral electrodes, and Pe is a positive-going wave following ERN, appearing about 200–400 ms after errors over parietal electrodes. Interestingly, studies using functional magnetic resonance imaging (fMRI) describe similar activations in the medial prefrontal areas for self-generated and observed errors^13^,^31^, suggesting that other- and self-generated errors involve a similar neural basis. Moreover, other studies using EEG reveal that similar observer ERN (oERN) and observer Pe (oPe) have been recorded in the prefrontal and parietal brain regions when participants observe others' errors^9^,^11^,^13^,^28^. Of note, the oERN and oPe are smaller in amplitude and can happen with a delay. According to previous studies, the most negative peak of oERN appears in the 200–350 ms time window^9^,^11^,^29^, and the most positive peak of oPe appears in the 250–500 ms time window^13^,^28^.

EEG rhythmic oscillations are always detected using spectral analysis methods such as the fast Fourier transform and wavelet transform. The spectral analysis can transform the single-trial time series to its oscillatory power, thus the neural activities which are treated as “background noise” in the ERP analysis can be shown as rhythmic oscillations within specific frequency bands in the time-frequency analysis, such as alpha (8–14 Hz) band^30^,^31^,^32^,^33^. It has been suggested that alpha band can be considered as a neural indicator of mental alertness or arousal^30^,^34^, behaving as reduced alpha power following the warning cue. Additionally, a growing number of studies have suggested alpha band reflects the modulation of attentional allocation^35^,^36^,^37^,^38^ and attentional orienting^39^.

In the present study, with the observation-execution Flanker task, we aimed to examine how the outcome valence and outcome frequency influenced PES and the associated ERP components (oERN and oPe), and the oscillatory band (alpha band). To address above issue, we varied level of outcome frequency as 50% and 80% (50/50 and 20/80, correct/error) in the observation task, which were counterbalanced between blocks. Before the experiment, participants were informed that their partners carry out the same task in another room outside the experimental chamber. Moreover, they could see the responses of each other on their own computer screens. This explicit instruction was used to ensure participants' engagement in the observation task. Actually, all partners were virtual and their responses were simulated, allowing that error rates of partners could be manipulated according to experimental purpose. In this case, the influence of outcome frequency was controlled in the 50% error rate condition, since the outcome frequency was equal between error and correct trials; whereas in the 80% error rate condition, infrequent correct trials only contained the information of outcome frequency.

Cognitive control account assumes that PES is driven by the error signal itself. Based on this view, we predict that PES should be observed both in the 50% and 80% error rate conditions. Conversely, orienting account considers that the slowing effect is driven by the infrequent outcomes. PES is just a special case of orienting response. Accordingly, in the 50% error rate condition, the reaction time (RT) in trials following errors and following correct trials should be comparable; in the 80% error rate condition, the slowing effect following infrequent correct trials should be observed.

The ERN and Pe components are typically evoked by the execution of error action, and one of the main functional roles of alpha band is to set and modulate brain attentional states^22^,^38^,^39^. Moreover, the study of HajiHosseini and Holroyd^22^ have revealed that ERP component reflects a reinforcement learning signal, whereas the time-frequency power exhibits greater sensitive to outcome probability. Thus, we predicted that the effect of outcome valence might be reflected in the oERN and oPe components, with larger activity following errors; the effect of outcome frequency may reflect in the alpha band, behaving as reduced power following infrequent correct trials.

## Results

### Behavioral results

Trials in which participants made wrong judgments in the observation task and/or made wrong responses in the execution task were discarded. Correct trials following errors in the execution task were also removed to rule out the confusion that the slowing in trials was due to the participants' own errors. Overall, 6.02% of trials were excluded.

For the analysis of PES, the results of repeated-measures analysis of variance (ANOVA) showed a main effect of outcome valence [correct trials following observation errors or following observation correct responses (eC and cC)], indicating that significantly slower RT on eC trials (844 ± 20 ms) than cC trials (829 ± 20 ms; *F*_1, 23_ = 14.23, *p* = 0.001). The main effect of outcome frequency (50% and 80% error rate conditions) was marginally significant (*F*_1, 23_ = 3.91, *p* = 0.06), showing that slower RT in the 50% (840 ± 20 ms) than in the 80% (833 ± 20 ms) error rate condition. Importantly, the two-way interaction was significant (*F*_1, 23_ = 4.57, *p* = 0.043). Post hoc tests revealed that RT on eC trials was slower than cC trials was observed for both the 50% (845 ± 20 ms vs. 835 ± 20 ms; *F*_1, 23_ = 4.54, *p* = 0.044) and the 80% (843 ± 20 ms vs. 823 ± 20 ms; *F*_1, 23_ = 22.35, *p* < 0.001) error rate conditions, meaning that PES effect was obtained both in the two error rate conditions (Fig. 2).

Regarding the accuracy of post-observation responses, all data in the execution task were used to analyze the effect, including the trials that were used as outlier trials (the correct trials following errors and errors in the execution task) in the analysis of PES. The results of the ANOVA did not reveal any significant results (*ps* > 0.20). In addition, to ensure that the participants really monitored their partners' performance, the accuracy of participants' oral reports were analyzed. The results of the ANOVA revealed a main effect for outcome valence (*F*_1, 23_ = 9.54, *p* = 0.005), indicating better performance on observation errors (95 ± 0.5%) than observation correct trials (93 ± 1.0%). And a main effect for outcome frequency (*F*_1, 23_ = 6.81, *p* = 0.016), indicating better performance on the 80% (95 ± 0.7%) than the 50% (93 ± 0.8%) error rate condition. However, the two-way interaction did not reach a significant level (*F*_1, 23_ = 1.06, *p* = 0.31).

### ERP results

*oERN*: The analysis of oERN focused on the midline sites (Fz, FCz, and Cz) where the effect was typically observed. Peak oERN amplitudes were submitted to the ANOVA with outcome valence (observation errors and observation correct responses) and outcome frequency as within-subject factors. The Greenhouse-Geisser correction was employed where appropriate. The main effects of outcome valence (*F*_1, 23_ = 2.29, *p* = 0.14) and outcome frequency (*F*_1, 23_ = 0.96, *p* = 0.34) were not significant. However, the two-way interaction did reach a significant level (*F*_1, 23_ = 4.45, *p* = 0.046). Post hoc tests revealed that the amplitude evoked by observation errors (−2.63 ± 0.50 μV) was significantly larger than that evoked by observation correct responses (oCRN; −2.07 ± 0.46 μV) only in the 50% error rate condition (*F*_1, 23_ = 4.32, *p* = 0.049) (Fig. 3a and Fig. 4a).

Further, since the scalp distribution of difference waveforms (error-correct) showed evident hemispherical effect for oERN (Fig. 3c), we conducted a three-way ANOVA to examine this hemispherical effect, with brain region (left-frontal and right-frontal), outcome valence and outcome frequency as within-subject factors. The results revealed that a main effect of brain region (*F*_1, 23_ = 6.53, *p* = 0.018), indicating that neural activity was significantly stronger on the left-frontal region (−2.06 ± 0.29 μV) than on the right-frontal region (−1.58 ± 0.35 μV) and two interactions of two factors (brain region by outcome valence: *F*_1, 23_ = 7.22, *p* = 0.013; response type by outcome frequency: *F*_1, 23_ = 5.18, *p* = 0.032). Post hoc tests revealed oERN (−1.81 ± 0.35 μV) was larger than oCRN (−1.35 ± 0.37 μV) in the right-frontal region (*F*_1, 23_ = 4.61, *p* = 0.042), but the effect was not found in the left-frontal region (*F*_1, 23_ = 0.32, *p* = 0.579) (Fig. 3b and Fig. 4b).

*oPe*: Peak oPe amplitudes were examined in the cento-parietal region (CP1, CP2, and CPz). Results showed that the main effects of outcome valence (*F*_1, 23_ = 1.95, *p* = 0.176) and outcome frequency (*F*_1, 23_ = 0.12, *p* = 0.735) were not significant. However, the two-way interaction was significant (*F*_1, 23_ = 3.87, *p* = 0.041). Post hoc tests revealed oPe amplitudes on observation correct responses (2.56 ± 0.44 μV) were larger than that on observation errors (1.94 ± 0.41 μV) only occurred in the 50% error rate condition (*F*_1, 23_ = 4.91, *p* = 0.037) (Fig. 3a and Fig. 4a).

### Time-frequency results

Based on the scalp regions showing most pronounced *F* values, two spatial regions of interest (S-ROIs) left-central region [(F1 + F3 + F5 + FC1 + FC3 + FC5 + C1 + C3 + C5)/9] and right-central region [(F2 + F4 + F6 + FC2 + FC4 + FC6 + C2 + C4 + C6)/9] were defined (in rectangles in Fig. 5b, *F* > 3.0, FDR corrected). In the above two regions, a time–frequency regions of interest (TF-ROI) alpha band (8–11 Hz, 650–900 ms) showed that the most pronounced task-related effect was defined (in rectangles in Fig. 5a, *p* < 0.05, FDR corrected).

The ERSP magnitudes within defined S-ROIs for each condition were entered into the two-way ANOVA in the left-central and right-central regions, respectively (Fig. 6). For the left-central region, the results showed the significant main effects of outcome frequency (*F*_1, 23_ = 4.18, *p* = 0.042) and outcome valence (*F*_1, 23_ = 5.55, *p* = 0.027). Moreover, the two-way interaction was significant (*F*_1, 23_ = 14.21, *p* = 0.001). Post hoc tests revealed that ERSP magnitudes on observation correct responses were significantly smaller in the 80% (−5.0 ± 2.5 ER%) than in the 50% (3.9 ± 2.3 ER%) error rate condition (*F*_1, 23_ = 15.77, *p* = 0.001), but that this was not case for observation errors (*F*_1, 23_ = 0.18, *p* = 0.677). For the right-central region, the results showed a similar effect. The main effects of outcome frequency (*F*_1, 23_ = 6.31, *p* = 0.019) and outcome valence (*F*_1, 23_ = 6.23, *p* = 0.02) were significant. Moreover, the two-way interaction was significant (*F*_1, 23_ = 8.56, *p* = 0.007). Post hoc tests revealed that the ERSP magnitudes on observation correct responses were significantly smaller in the 80% (−5.2 ± 2.4 ER%) than in the 50% (1.3 ± 1.6 ER%) error rate condition (*F*_1, 23_ = 10.63, *p* = 0.003), but that this was not case for observation errors (*F*_1, 23_ = 0.01, *p* = 0.911).

### Correlation analysis results

The results showed that the average of the regression coefficients for the oPe (−0.004 ± 0.015; *t*_23_ = −1.34, *p* = 0.193) and the alpha (−0.08 ± 0.23; *t*_23_ = −1.71, *p* = 0.101) did not significantly differ from zero, whereas for the oERN (0.006 ± 0.015; *t*_23_ = 1.95, *p* = 0.043), the result showed a significant positive correlation between oERN amplitude and PES, which confirmed that increased oERN amplitude predicted increased RT of post-error trials.

## Discussion

In the present study, we examined how outcome valence and outcome frequency influenced PES from the EEG perspective, including ERP and time-frequency analyses. The behavioral results showed that slower RT on correct trials following observation errors than following observation correct responses both in the 50% and 80% error rate conditions, reflecting that significant PES was observed in the two conditions. ERP analysis revealed that the difference activity between observation errors and observation correct responses only occurred in the 50% error rate condition for both oERN and oPe. However, time-frequency analysis revealed the inverse resluts. The difference activity between the two response types only occurred in the 80% error rate condition for alpha band. More importantly, the correlation analysis displayed a significantly positive correlation between oERN and PES. These findings suggest that error signals and infrequent events are both involved in post-error behavioral adjustment but are associated with different neural processes.

RT on correct trials following observation errors tended to be slower than that following observation correct responses in both 50% and 80% error rate conditions, fitting with the assumation of cognitive control account^2^. It implies error signal per se generate the PES effect. Meanwhile, faster rather than slower responses on correct trials following infrequent observation correct responses occurred in the 80% error rate condition, suggesting that attention process induced by infrequent outcomes is also involved in the generation of PES. However, the attention process does not reflect attention orienting to “surprising” signals. Regarding the accuracy, although participants performed better for error trials than for correct trials in the observation task, there was no difference between post-observation error and post-observation correct accuracy. Current result pattern cannot fulfill the expectation of the orienting account^5^, because this account considers that the performance is worse after errors than after correct trials. Meanwhile, the accuracy did not improve following observation errors. It is likely that the ceiling effect influences the participants not to up-regulate their performances in the present study.

ERN and Pe are two typical components elicited by error commission, serving as indicators of activities of error monitoring system^21^,^26^. When the proportion for observing either an error or a correct response is equal, error signals may act as the uniquely valid cue that participants can use to adjust the subsequent performance. In accordance with this, current data showed that the difference of neural activity only occurred in the 50% error rate condition for both oERN and oPe. Further, the correlational results affirmed that oERN amplitude positively correlated with PES, paralleling with previous cognitive studies^2^,^21^,^40^. Together, these findings may suggest the ERP component is sensitive to the outcome valence^22^.

This conclusion was further corroborated by the hemispherical effect analysis of oERN (Fig. 3c and Fig. 4b): the most pronounced difference activity (error–correct) located in the right lateral prefrontal cortex, with stronger oERN amplitude in this region. Right lateral prefrontal cortex is part of the right hemispherical inhibition network, which has been associated with motor stopping or slowing^41^,^42^,^43^,^44^. In the present study, when the participants observed their partners' responses were inconsistent with their own responses, they might have produced a key-press impulse to rectify the error responses. However, before experiment, they were explicitly instructed not to make any press, just to observe their partners' responses in the observation task. In this case, participants needed to inhibit their key-press impulses, especially when the partners committed errors. Additionally, since increased activity in the right lateral prefrontal cortex on errors compared with that on correct responses reflects the degree of caution^42^, stronger oERN in the right-frontal region supports that error signals trigger enhanced cognitive control to minimize the risk of subsequent errors.

In particular, it is worth noticing that the process induced by error signals is also involved in the generation of PES in the 80% error rate condition. As shown in Fig. 3c, increased oERN amplitude in the right hemisphere also occurred in the 80% error rate condition, though its activity was weaker than that in the 50% error rate condition. Thus our results seem inconsistent with the orienting account^5^, including the behavioral and ERP results. There are likely multiple reasons why these findings among studies on post-error adjustment are inconsistent. First, all errors are not alike. Errors are caused by encoding failures at the time of the stimulus or by fast guessing in the study of Notebaert and his colleagues^5^, whereas errors are caused by comparing the representations of the correct response with the representations of the partners' actual response in the present study^17^. Recent studies have affirmed that error adjustments depend on the type of error^6^,^7^. Second, this may be linked to the task set. Notebaert and his colleagues achieve predetermined error rates by increasing the perceptual difficulty, which prompts participants to consume large resources to complete the stimulus discrimination in the high error rate condition. In this case, cognitive recourses are exhausted in the hard condition, resulting in no remaining recourses to detect and correct errors. Thus, they discover infrequent outcomes evoke the orienting response without error adjustment. However, we vary the levels of error rate without increasing task difficulty. Thus, participants still have cognitive resources to process and utilize the error information in the 80% error rate condition.

In relation to the time-frequency results, a totally reverse pattern was observed. It is highly conceivable that the alpha band is associated with mental arousal^30^,^34^ and attention process^35^,^36^,^37^,^39^. As an inverse neural indicator, increase in focusing attention or mental arousal will result in a relative decrease in power in the alpha band. For instance, alpha power reduces to the attended location and increases to the ignored location in a visual spatial attention task^36^,^37^. The correct responses of 80% error rate condition induced significantly reduced alpha band, reflecting increased attention or mental arousal on the infrequent correct responses. It may directly account for why faster responses on the correct trials following infrequent observation correct responses in the 80% error rate condition. The enhanced selective attention leads to improved performance, supporting the view that attention processing induced by infrequent correct responses modulates PES in a top-down goal-directed way. The finding of Parmentier, Elsley, and Ljungberg^45^ further supports this result, arguing that when the cognitive system make use of the infrequent events as valid warning cues, the frequency information can be used for the goal-relevant purpose, and facilitation instead of distraction will be observed. Moreover, the difference of oscillatory power only occurred in the correct trials between the two error rate conditions, which resulted in the difference of outcome valence only occurred in the 80% error rate condition (Fig. 6). Since infrequent correct trials in the 80% error rate condition only contain the information of outcome frequency, this result indicates that the alpha band is sensitive to the outcome frequency.

Furthermore, studies investigating the association between neural indicators and PES have reported equivocal results. Some consider that error signals can predict the extent of PES, behaving as PES increases with ERN amplitude^21^,^40^; whilst others find that attentional orienting rather than error evaluation predicts PES, showing Pe or feedback-related positivity triggers PES^8^,^20^. More recently, the study of Carp and Compton^30^ suggests that alpha band associated with attention process is a better indicator of individual difference in PES than ERN amplitude. However, in the present study, the correlation analysis revealed that oERN amplitude was positively correlated with PES, according with the results of previous cognitive studies. This result directly illustrates that outcome valence determines the generation of PES.

In addition, based on the illustration of Ridderinkhof^4^, macro-adjustment involves the long-term strategic modulation in response to the relative probability of trials, while micro-adjustment involves event-by-event modulation invoked by the commission of incidental errors. ERP components are time-locked to the events of interest, and modulated by the transient responses; whereas rhythmic oscillations reflect the state of the processing network, the modulation is likely to remain constant for some time^46^,^47^. Therefore, the strategic modulation of outcome valence or outcome frequency reflects on a micro- or macro-level, respectively.

In conclusion, with observation-execution Flanker task, PES was observed in the two (50% and 80%) error rate conditions, in particular, faster rather than slower responses on the infrequent correct trials were observed in the 80% error rate condition, fitting with the assumption of the cognitive control account. However, oERN and oPe components and alpha power were differentially sensitive to outcome valence and outcome frequency, paralleling the HajiHosseini & Holroyd study^22^. For the ERP components, the difference of neural activity between observation errors and observation correct responses only occurred in the 50% error rate condition; whereas for the alpha band, this difference only occurred in the 80% error rate condition, suggesting that outcome valence and outcome frequency were both involved in PES. Most importantly, the correlational analysis revealed oERN amplitude was positively correlated with PES, affirming that outcome valence plays a crucial role in the generation of PES (modulation on a micro-level), but outcome frequency modulates the extent of PES (modulation on a macro-level).

## Methods

### Subjects

Twenty-six healthy volunteers (eighteen females, all right-handed with normal or corrected-to-normal vision, aged 18–24 years) took part in the experiment for payment. Two participants had to be excluded for the bad EEG record (too many artifacts). Finally, the data from 24 participants (sixteen females) were used to the behavioral and EEG analyses. Each participant signed written informed consent and was unaware of the purpose of the experiment. The study was in accordance with the Declaration of the Southwest University (SWU) Brain Imaging Center Institutional Review Board and approved by the ethics committee of SWU.

### Apparatus and Stimuli

The experiment was run using E-Prime software (Psychology Software Tools, Inc. Pittsburgh, PA) displayed on a 17-inch monitor of a Lenovo computer (with a refresh rate of 85 Hz and a resolution of 1024 by 768). The stimuli of the flanker task were four capital English letters (H, N, E, and R), which were presented in black on a grey background. In congruent trials, the central letter and the flankers were the same, e.g. HHHHH; in incongruent trials, the central letter and the flankers were different, e.g. HHNHH. Congruent and incongruent trials were pseudo-randomly sequenced with equal frequency. Participants were instructed to respond to the central letters. The letter-response key mappings were 1, 2, 9, and 0 on horizontally-arranged number keys on a standard keyboard. Specifically, the four target letters (H, N, E, and R) were mapped on the 1 key (left middle finger), 2 key (left index finger), 9 key (right index finger), and 0 key (right middle finger), respectively.

### Experimental procedure

**Figure 1:**
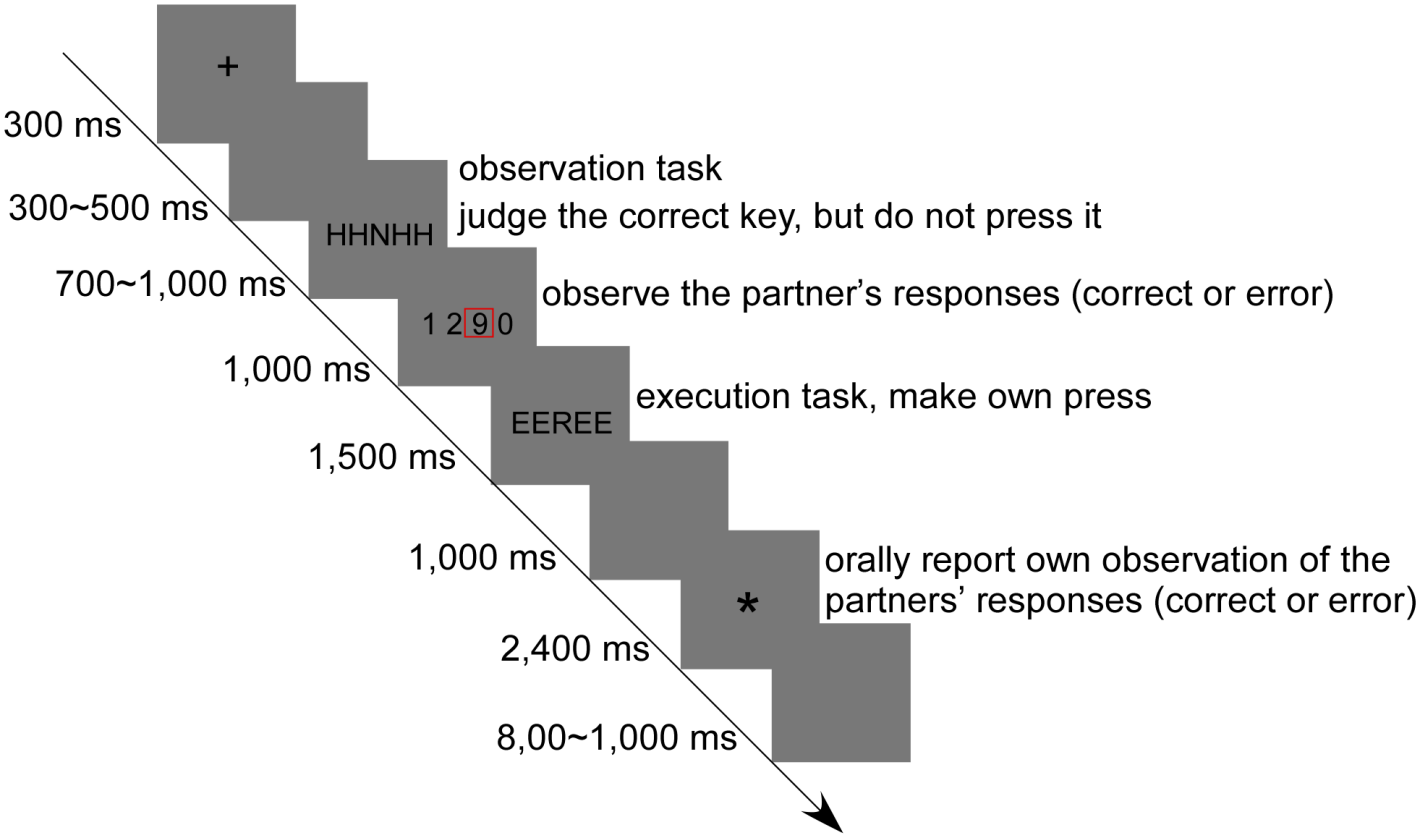
Illustration of the time parameters and task sets of one trial. In each trial, participants first viewed the outcome of a flanker-run that was supposedly perform by a “partner” in another room, and then performed a flanker-run themselves afterwards. The first array of five letters was the observation Flanker task, in which participants just needed to observe but do not overtly press any keys; the second array of five letters was the execution Flanker task, in which participants needed to make own press according to the mapping rules. The number in the red box indicates the answer of the virtual partner.

**Figure 2:**
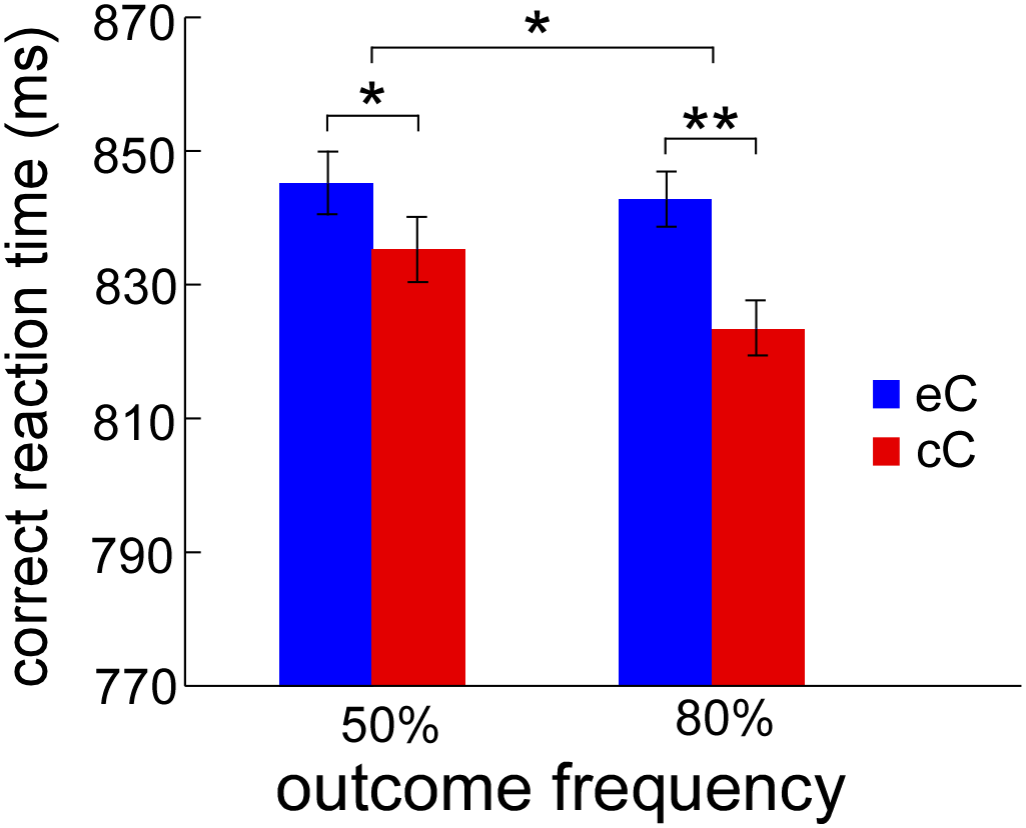
The mean reaction time of the execution task for eC and cC. The reaction time on eC is significantly larger than that on cC for both 50% and 80% error rate conditions, indicating PES is obtained both in the two error rate conditions. eC (blue bars) represents the mean RT on correct trials following observation errors, and cC (red bars) represents the mean RT on correct trials following observation correct responses. ms means milliseconds. Error bars denote standard error. Significant differences are indicated by asterisks (* *p* < 0.05, ** *p* < 0.01).

The experimental timing of one trial is illustrated in Fig. 1. The participants comfortably sat in a soundproof room at a distance of approximately 60 cm from the screen. Each trial started with a 300 ms cross fixation, followed by a 300–500 ms blank screen. The observation task was then presented. An array of five letters was randomly presented in the center of the screen for 700–1,000 ms, and participants mentally made a response to the central letter but did not overtly make a key-press. Following that, the response set (four numbers) was presented on the screen for 1,000 ms with one surrounded by a red outline, which indicated the virtual partners' response. Here, the participants needed to judge the correctness of the responses made by their partners and remember their judgments, which they were required to report orally at the end of each trial. After completing the observation task, participants needed to perform an execution task. A stimulus was presented for a maximum of 1,500 ms (and terminated after any response key was pressed within this interval), during which period participants were required to press the corresponding key as quickly and accurately as possible. After the stimulus disappearance, a blank screen was displayed for 1,000 ms. Next, an asterisk cue reminded participants to orally report their judgments about the virtual partners' responses, which were recorded by the experimenter through a serial response box (SRBOX). The asterisk cue sign was terminated by a key press within 2,400 ms. Finally, a blank screen was displayed for 800 to 1,000 ms.

Each participant completed a practice block of 50 trials prior to completing eight experimental blocks (80 trials per block, 640 trials in all), with a one minute break between blocks. Only when accuracy in the practice block exceeded 80% could participants go on to perform the formal experiment. In addition, completing each block, the key-press accuracy in the execution task was presented for 1,000 ms to maintain the participants' attention.

### EEG data collection

The EEG data were recorded using a 64-channel Brain Products system (Brain Products GmbH, Germany; passband: 0.01–100 Hz, sampling rate: 500 Hz) that was connected to a standard EEG cap based on the extended 10–20 system. All signals were referenced to the left mastoid but were later off-line re-referenced to the average of both mastoids. The vertical electrooculogram (EOG) was recorded bipolarly from electrodes placed above and below the right eye. The horizontal EOG was also recorded bipolarly from electrodes lateral to both eyes. All channel impedances were kept below 5 kΩ. Trials contaminated with mean EOG artifacts exceeding ± 100 μV or those with artifacts due to amplifier clipping, bursts of electromyographic (EMG) activity, or peak-to-peak deflection exceeding ± 100 μV were excluded from averaging. Data were filtered offline with a passband 0.1–20 Hz^48^.

### ERP analysis

**Figure 3:**
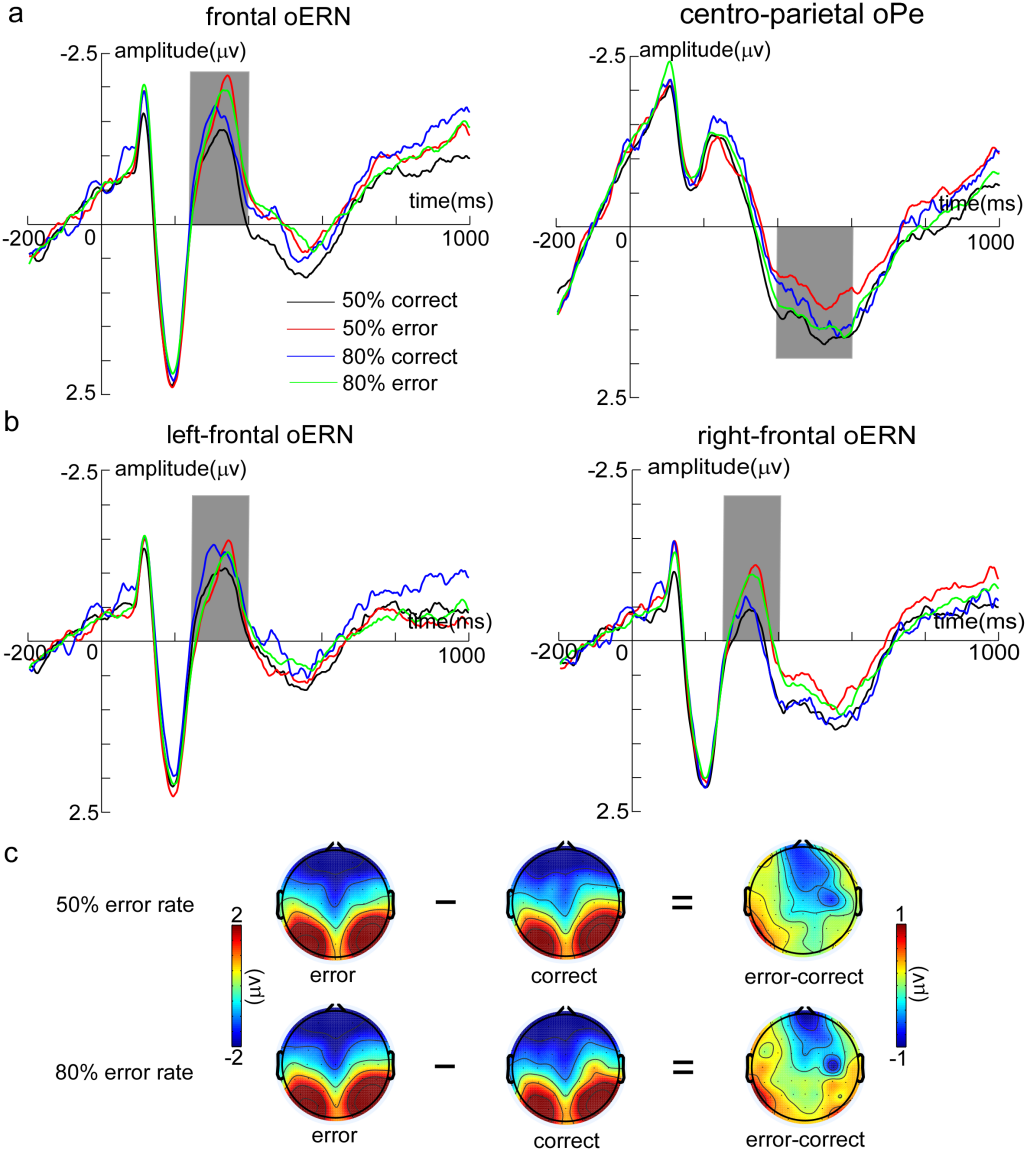
The ERP results in the observation task. (a) The response-locked grand-averaged ERP waveforms for each condition (50% correct, 50% error, 80% correct, and 80% error) in the frontal region [(Fz + FCz + Cz)/3] and centro-parietal region [(CP1 + CP2 + CPz)/3] for oERN and oPe, respectively. (b) The hemisphere effect analysis in the defined time window for oERN, including left-frontal region [(F1 + F3 + FC1 + FC3 + C1 + C3)/6] and right-frontal region [(F2 + F4 + FC2 + FC4 + C2 + C4)/6]. The neural activities in the defined time windows are presented in the grey bar for each region (oERN: 250–400 ms; oPe: 400–600 ms). 50% correct (black line) represents the neural activity of observation correct responses in the 50% error rate condition, 50% error (red line) represents the neural activity of observation errors in the 50% error rate condition, 80% correct (blue line) represents the neural activity of observation correct responses in the 80% error rate condition, and 80% error (green line) represents the neural activity of observation errors in the 80% error rate condition. (c) The topography distributions of two response types (observation error and observation correct response) and the difference between the two for 50% and 80% error rate conditions, respectively.

**Figure 4:**
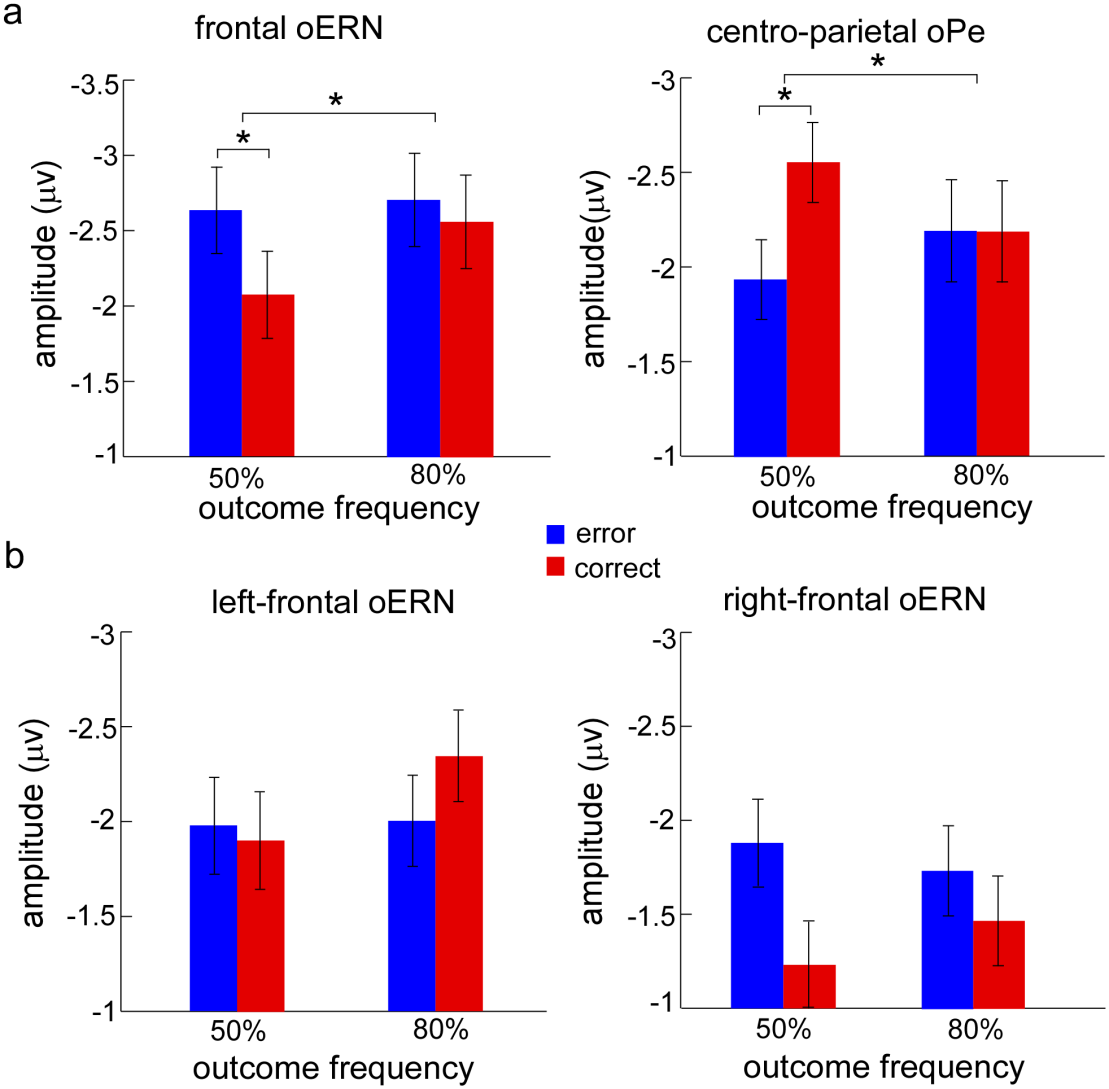
The mean amplitude in the observation task for observation errors and observation correct responses in the 50% and 80% error rate conditions, respectively. (a) The neural activity evoked by observation errors and by observation correct responses in the 50% and 80% error rate conditions for frontal oERN and centro-parietal oPe. (b) The hemisphere effect of oERN between left-frontal and right-frontal regions. error (blue bars) represents the neural activity evoked by observation errors, and correct (red bars) represents the neural activity induced by observation correct responses. Error bars denote standard error. Significant differences are indicated by asterisks (* *p* < 0.05).

ERPs analyses were time-locked to the onset of the partners' responses and averaged separately for observation correct trials and observation errors relative to a 200 ms pre-observation response baseline. oERN amplitude was defined as the most negative peak in the 250 to 400 ms time window at frontal region [(Fz + FCz + Cz)/3]. oPe amplitude was defined as the most positive peak in the 400 to 600 ms time window at centro-parietal region [(CP1 + CP2 + CPz)/3]. In addition, based on the scalp topography distributions of the difference activity (error-correct) in the analysis time window for oERN (Fig. 3c), left-frontal [(F1 + F3 + FC1 + FC3 + C1 + C3)/6], and right-frontal [(F2 + F4 + FC2 + FC4 + C2 + C4)/6] regions were also defined as the region of interest for examining the hemispherical effect of oERN.

### Time-frequency analysis

The preprocessing of time-frequency analysis was conducted by Brain Vision Analyzer 2.0 (Brain Products GmbH, Germany) and EEGLAB (an open source toolbox running in the MATLAB environment for EEG signal processing)^24^. First, as the analysis of ERP components, resetting new reference, removing EOG artifacts, extracting epochs and correcting baseline were completed in the analyzer 2.0. Of note, considering that the relatively low time resolution for the time-frequency analysis, we chose a relatively long baseline to get a steady estimation for low frequencies (e.g., alpha band). Thus EEG data were segmented into a time window from −500 to 1,000 ms that was time-locked to the observation response and baseline corrected using the interval of pre-observation response (−500 to 0 ms). Then, data were imported into EEGLAB. Remaining artifacts in the EEG were further addressed using a ±100 μV threshold by EEGLAB, and corresponding epochs were excluded. Following that, the resulting data were transformed into the time-frequency domain using continuous Morlet wavelet transform (CWT) conducted by Letswave software (http://amouraux.webnode.com)^23^. After completing all of the EEG preprocessing, an estimate of the oscillatory power as a function of time and frequency (time-frequency representation) was obtained from single-trial EEG epochs by CWT. The parameters of central frequency (ω) and restriction (σ) in CWT were 5 and 0.15 respectively, and time-frequency representations were explored between 1 to 20 Hz in steps of 0.58 Hz. Single-trial time-frequency representations were then averaged to obtain averaged time-frequency representations of every participant under each condition^32^,^33^. Subsequently, the averaged time-frequency representations were exported from Letswave and imported into MATLAB for further analysis.

**Figure 5:**
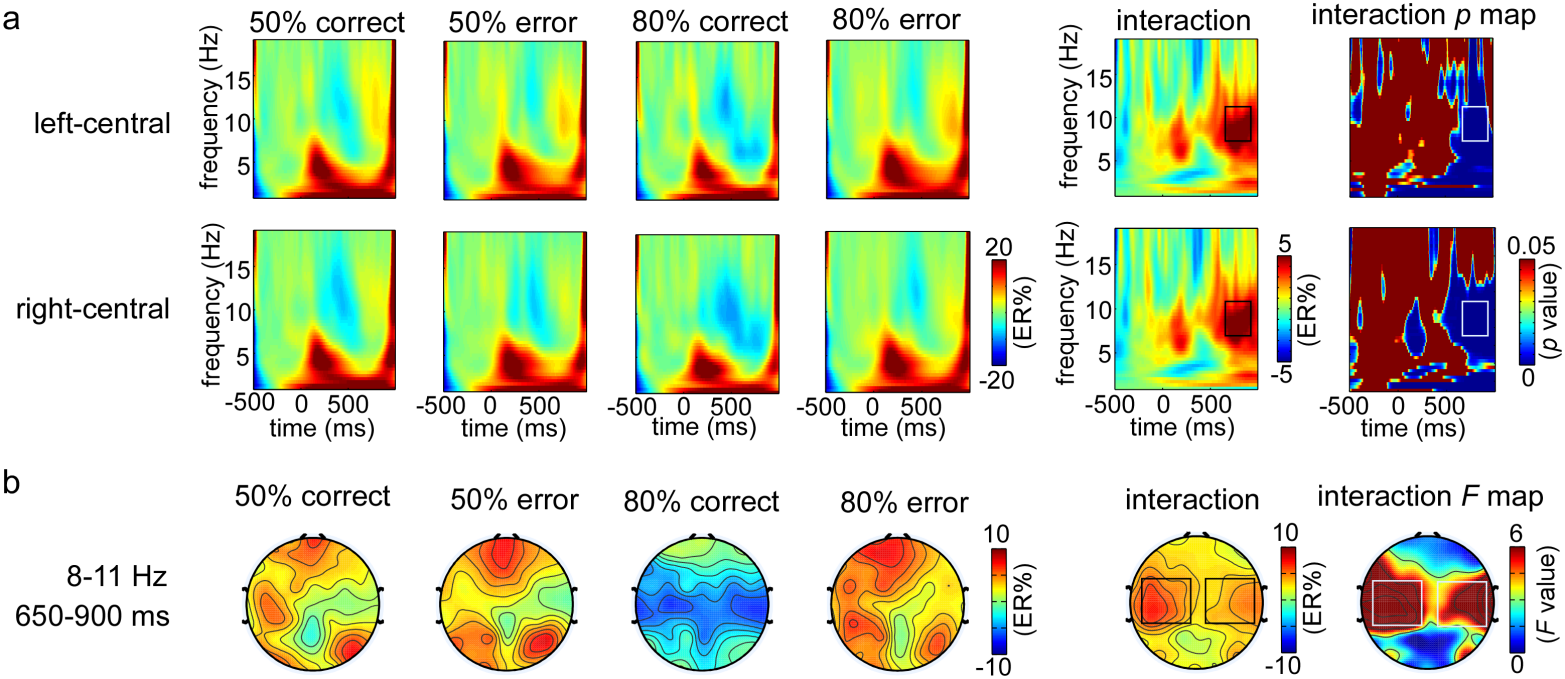
The time-frequency resluts in the observation task. (a) The grand-average time-frequency representations (expressed as ER%) for each condition (50% correct, 50% error, 80% correct, and 80% error) and the interaction between outcome valence and outcome frequency within the defined S-ROIs, including left-central region [(FC1 + FC3 + FC5 + C1 + C3 + C5 + CP1 + CP3 + CP5)/9] and right-central region [(FC2 + FC4 + FC6 + C2 + C4 + C6 + CP2 + CP4 + CP6)/9]. The corresponding interaction *p* map is the reslut of bootstrapping statistical analysis at the significance level of *p* < 0.05 (FDR corrected), which is used to define the TF-ROI in each S-ROI. Note that a pre-response interval from −450 to −50 ms is used as the baseline. The time–frequency pixels displaying a significant difference from the baseline are colored in blue. The significant task-related TF-ROIs are outlined in the rectangles. Each row corresponds to one S-ROI corresponding to the largest modulation of the task-related effects. X-axis, time (ms); Y-axis, frequency (Hz). (b) The scalp topographies of ERSP magnitudes for each condition and the two-way interaction within the defined TF-ROI (alpha band). The corresponding interaction *F* topography is the reslut of two-way repeated-measures ANOVA, which is used to define the significant S-ROI in the corresponding TF-ROI. 50% correct represents the neural activity of observation correct responses in the 50% error rate condition, 50% error represents the neural activity of observation errors in the 50% error rate condition, 80% correct represents the neural activity of observation correct responses in the 80% error rate condition, and 80% error represents the neural activity of observation errors in the 80% error rate condition.

**Figure 6:**
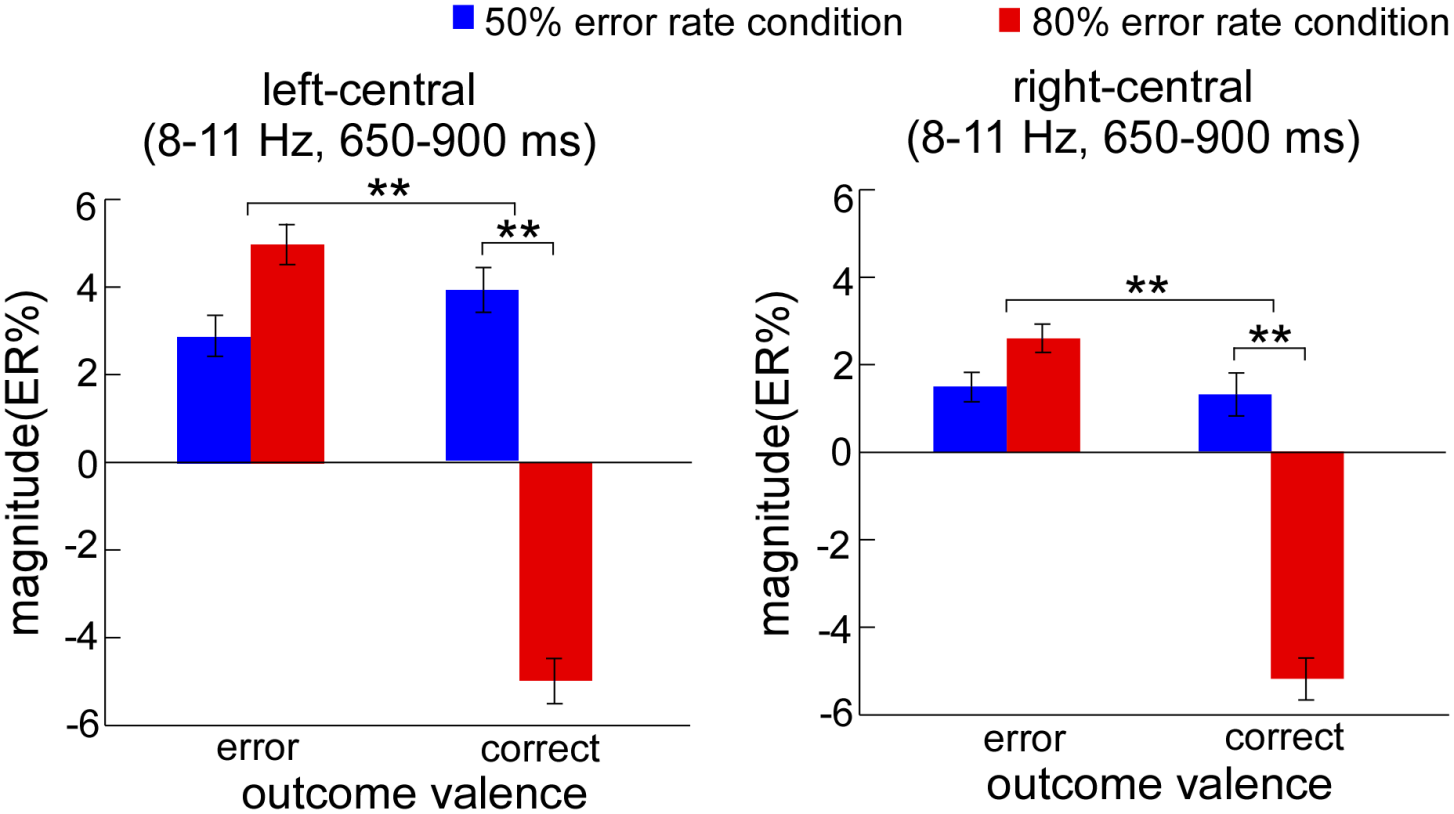
The mean ERSP magnitudes (expressed as ER%) for the alpha band (8–11 Hz, 650–900 ms) in the observation task. ERSP magnitudes on observation correct responses were significantly smaller in the 80% than in the 50% error rate condition for left-central and right-central regions. The blue bars represent the neural power induced by observation responses in the 50% error rate condition. The red bars represent the neural power induced by observation error responses in the 80% error rate condition. ms means milliseconds. Error bars denote standard error. Significant differences are indicated by asterisks (** *p* < 0.01).

To analyze the power modulation of ongoing EEG rhythms after the onset of observation responses, an event-related spectral perturbation (ERSP) was calculated for every time-frequency pixel in the time-frequency representations. In each specified frequency band, ERSP was displayed as a transient increase (event-related synchronization, ERS) or decrease (event-related desynchronization, ERD) in oscillatory power relative to the baseline interval according to the following formula: ER_t,f_ % = [A_t,f_ - R_f_]/R_f_, where A_t,f_ was the signal power at a given time (t) and frequency (f), and R_f_ was the signal power averaged within the baseline interval^49^. To avoid edge effects when performing CWT, pre-observation response −450 to −50 ms was used as the baseline interval in the present study. After transforming the original power to ERSP in the time–frequency representations, we performed an exploratory data-driven approach to identify all the spatial regions of interest (S-ROIs) and time–frequency regions of interest (TF-ROIs). According to the *F* map of interaction between outcome valence and outcome frequency, two S-ROIs which were related to the most pronounced modulations were identified: left-central region [(F1 + F3 + F5 + FC1 + FC3 + FC5 + C1 + C3 + C5)/9] and right-central region [(F2 + F4 + F6 + FC2 + FC4 + FC6 + C2 + C4 + C6)/9] (Fig. 5b). Additionally, according to the *p* map of interaction, the maximal time-frequency power and corresponding peak power latencies were chosen as TF-ROI. Since TF-ROI had to be composed of more than 75 consecutive significant time points (>150 ms)^50^, alpha band (8–11 Hz in frequency, 650–900 ms in latency) was defined as TF- ROI (Fig. 5a).

### Correlation analysis

To directly investigate the relationship between the neural indicators (oERN, oPe, and alpha) and behavioral slowing effect, we performed the regression analyses of repeated measures data^8^,^51^. First, separate regression equations were computed for each participant. According to our experimental design, for each participant, we had four values for each neural indicator and four RT measures. The neural indicator values were the average peak measure of a participant in one condition (50% error, 50% correct, 80% error, and 80% correct). The four RT measures were the participants' mean RTs for correct trials following this particular condition. Thus the four RT measures were regressed on the oERN, oPe, and alpha, where three predictors were entered simultaneously into the equation. After the regression equations were computed, we acquired three regression coefficients of neural indicators for each participant. Second, we tested each regression coefficient to see whether it differed reliably from zero.

## Author Contributions

L.J.W. and A.T.C. conceived and designed the experiment. L.J.W., Y.F.Z., S.S.W. and J.F.T. performed the experiment. L.J.W. and A.T.C. analyzed EEG recordings. L.J.W., D.D.T. and A.T.C. wrote the manuscript. H.G. contributed to refine the language.
